# Untargeted metabolism approach reveals difference of varieties of bud and relation among characteristics of grafting seedlings in *Camellia oleifera*

**DOI:** 10.3389/fpls.2022.1024353

**Published:** 2022-11-21

**Authors:** Wei Long, Guangyuan Huang, Xiaohua Yao, Leyan Lv, Chunlian Yu, Kailiang Wang

**Affiliations:** ^1^ Zhejiang Provincial Key Laboratory of Tree Breeding, Research Institute of Subtropical Forestry, Chinese Academy of Forestry, Hangzhou, Zhejiang, China; ^2^ Chang Country Oil Tea Industry Development Center, Changshan Country Bureau of Forestry & Water Resoures, Changshan, Zhejiang, China; ^3^ College of Hydraulic Engineering, Zhejiang Tongji Vocational College of Science and Technology, Hangzhou, Zhejiang, China

**Keywords:** *Camellia oleifera*, ultra-performance liquid chromatography/mass spectrometry, scion, bud, utargeted-metabolomics, metabolites

## Abstract

*Camellia oleifera* is one of the essential wood oil trees in the world. *C.oleifera* was propagated by nurse seedling grafting. Since the scion of *C.oleifera* had a significant regulated effect on the properties of rootstock after grafting and impacted on the growth of the grafted seedlings, it was necessary to understand the characteristics of buds among varieties to cultivate high-quality grafted seedlings. The metabolome was thought to be a powerful tool for understanding connecting phenotype-genotype interactions, which has an important impact on plant growth and development. In this study, UPLC-MS was used to determine the metabolites of the apical buds of CL3, CL4, CL40, and CL53 spring shoots after 30 days of sprout and to measure the growth characteristics of roots and stems after grafting. Metabolomics analysis revealed 554 kinds of metabolites were significant differences among four varieties, and 29 metabolic pathways were identified to have significant changes (*p<* 0.05), including carboxylic acids and derivatives, fatty Acyls, organooxygen compounds, and prenol lipids metabolites. The metabolites appeared in all varieties, including phenethyl rutinoside in glycosyl compounds and hovenidulcioside A1 in terpene glycosides. Metabolite–metabolite correlations in varieties revealed more complex patterns in relation to bud and enabled the recognition of key metabolites (e.g., Glutamate, (±)Catechin, GA_52_, ABA, and cs-Zeatin) affecting grafting and growth ability. Each variety has a unique metabolite type and correlation network relationship. Differentiated metabolites showed different growth trends for development after grafting. Many metabolites regulate the growth of scions in buds before grafting, which plays a crucial role in the growth of seedlings after grafting. It not only regulates the growth of roots but also affects the development of this stem. Finally, those results were associated with the genetic background of each cultivar, showing that metabolites could be potentially used as indicators for the genetic background, indicating that metabolites could potentially be used as indicators for seedling growth characteristics. Together, this study will enrich the theoretical basis of seedling growth and lay a foundation for further research on the molecular regulation mechanism interaction between rootstock and scion, rootstock growth, and the development of grafted seedlings after grafting.

## Introduction

As an essential woody oil tree species in southern China (Zhuang, 2008), *C.oleifera* has 4.53 million hectares (Zhang and Wang, 2021). The oil obtained from its seeds after pressing in *C.oleifera* is favored by consumers. With the increasing oil demand, its planting area continues to expand, and varieties have been introduced to many places (Yang et al., 2011; Yan et al., 2012; Du et al., 2013; Wang et al., 2013; Bu et al., 2015), becoming an essential helper for the vast forest areas to get rid of poverty and become rich. So it improved the growing demand for elite seedlings. The seedlings of *C.oleifera* are often bred by grafting. In practice, it was found that there was apparent rootstocks-scions interaction in grafting seedlings of *C.oleifera*, and root growth after grafting was controlled by scions (Long et al., 2013). There were significant differences in tree potential and growth among varieties (Cao et al., 2014; Li et al., 2020). These phenomena indicated that the branch position of the scions significantly affected the growth of the root and shoots of the grafted seedlings. Therefore, understanding the characteristics of the scion buds to regulate the growth mechanism of grafted seedlings was very important to realize the efficient cultivation of seedlings.

The effect of rootstocks-scions interaction had been widely found in agriculture (Gautier et al., 2019; Rasool et al., 2020; Tsaballa et al., 2021). The three critical components of graft, including rootstock, scion, and grafting union, played a crucial role in the interaction (Gautier et al., 2019; Rasool et al., 2020). The rootstocks regulated the absorption of nutrients in rhizosphere soil, rhizosphere microbial communities, and the transportation of water, nutrients, and other substances. They changed the scion phenotype and resistance by the exchange of hormones, proteins, and small RNAs between rootstock and scion (Tsago et al., 2014; Nimbolkar et al., 2016), resistance to diseases (Kumbar et al., 2021), and yield and quality (Santarosa et al., 2016), and seedling rootstock breeding (Cañas-Gutiérrez et al., 2022). The grafting union was the primary channel for the communication between rootstocks and scions, and the reconstruction and development of vascular bundles played a vital role in the communication between rootstocks and scions, thus affecting the development of scions (Adams et al., 2018; Santarosa et al., 2016; Tsaballa et al., 2021). It should be noted that there was less research on affecting scions to rootstocks than rootstocks to scions. However, this influence had long been recognized (Amos et al., 1930; Albacete et al., 2015; Warschefsky and Rieseberg, 2021). The effect of scions on rootstocks might significantly change the structure and growth of roots and rhizosphere microbial communities, including the systematic regulation of arbuscular mycorrhizal (AM) and root hair development (Zapata et al., 2001; Tandonnet et al., 2010; Callesen et al., 2016; Shu et al., 2017; Chai et al., 2022). In model species, there were many examples of bud signals regulating root development (Yoshida et al., 2021), such as metabolites, hormones, peptides, HY5 (Chen et al., 2016), microRNA 156 (Bhogale et al., 2014), miRNA172 (Martin et al., 2009) and microRNA 399 (Lin et al., 2014). This indicated that many substances were involved in regulating scion to rootstock after grafting. Therefore, it was necessary to do more research to understand how scions affect rootstocks.

Because many life activities in cells occur at the metabolite level, metabonomics could intuitively understand the phenotypic characteristics of plants by studying the changes of metabolites in the environment and the growth and development process. Unique metabolites played a crucial role in plant adaptation to the environment and resistance to biological and abiotic stresses (Fernández-Paz et al., 2021). Traditionally, plant metabonomics research had focused on clarifying the function and regulation of specific biosynthetic pathways involving many metabolites (Stitt et al., 2010; Fernie and Tohge, 2017). The metabolomic analysis was widely used to study critical agricultural traits, such as flavor, yield, biomass, and nutritional quality. It was confirmed that grafting significantly increases the content of primary metabolites in Cucumber (Liang et al., 2021) and watermelon fruits (Aslam et al., 2020; Zhang et al., 2022). In addition to biosynthesis and accumulation in a tissue-specific manner, metabolites could be produced and transported across tissues and organs to regulate biological processes (Van de Poel et al., 2014). The type and content of metabolites could directly control the differentiation and development of buds. Moreover, the growth state of scions could be reflected by analyzing the type and expression level of metabolites in buds (Dai et al., 2020). At present, the research on the regulation mechanism of *C.oleifera* growth and development was mostly based on physiological and transcriptional genes (Lin et al., 2018; Gong et al., 2020; Long et al., 2022), while metabonomics research usually focuses on seed development and dynamic oil changes in post-harvest stage and flower bud differentiation (Feng et al., 2014; Qiu et al., 2015; Xie et al., 2018; Fu et al., 2018; Huang et al., 2021).

These researches showed that the interaction between rootstock and scion significantly affected the root and stem growth, fruit quality, and yield of grafted seedlings. It indicated that there might be a key metabolite regulating root growth in the scion of *C.oleifera*. In this paper, we explore the types of metabolites contained in the bud of *C.oleifera* and the growing differences among varieties after grafting. It could provide more accurate cultivation strategies, improve seedlings’ reproductive efficiency and quality, and ultimately help achieve low-cost, efficient, and automated seedling raising of *C.oleifera*.

## Materials and methods

### Plant materials

Five-year-old trees of cultivars, including CL3, CL4, CL40, and CL53, were used in this research. The plants were grown in National Core Germplasm of *C. oleifera* in Dongfanghong Forestry, Jinhua City, Zhejiang Province. He and Yao, (2013) described the characteristics of varieties. The tree branches were defined from four directions in the middle of the tree, and the scions in branches were cut on the thirtieth day after sprouting (Long et al., 2019). The terminal buds of scions were collected and mixed. The samples were immediately frozen in liquid nitrogen and stored at -80 °C for subsequent UPLC/MS. Each test of each variety consisted of six biological replicates.

**Figure 1:**
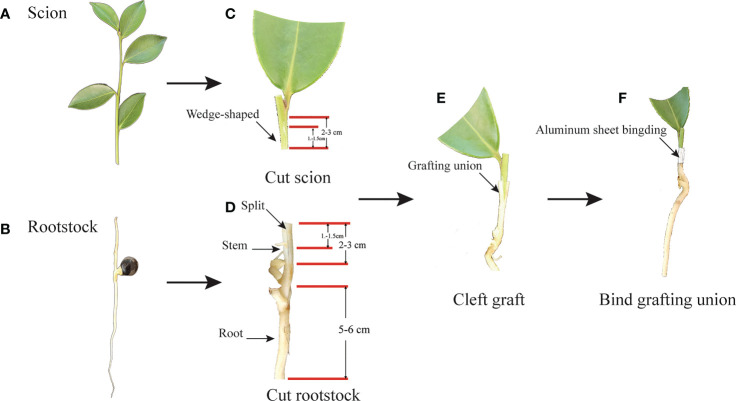
The process of nurse seedling grafting. **(A)** Scion: semi-wooden scion. **(B)** Rootstock: un lignified seedling rootstock of half-sib seed. **(C)** Cut scion: Cut semi-wooden scion (2-3cm long) from the same trees as rootstock seeds, and cut length in 1.2-1.5cm with wedge-shaped and meet pith. Then cut half the leaves of the scion. **(D)** Cut rootstock: cutting 2-3 cm from the stem of the seedling, and Split about 1.0-1.5 cm in the middle of the stem, and trim about 5-6 cm in the root of the rootstock. **(E)** Cleft grafting: the wedged-scion inserted into the split of rootstock. **(F)** Bind grafting union: fasten the aluminum sheet on the joint of the rootstock and the scion tightly combined.

Meantime, the buds of scions, including CL3, CL4, CL40, and CL53, were selected for grafting by nurse seedling grafting technology (Long et al., 2013; Feng et al., 2017) (**Figure 1**). Mature half-sib seeds of the varieties were collected from Dong fang hong Forestry, Jinhua City, Zhejiang Province, China, and stored in a sand bed for 2 months. The seeds were tiled in sand bed with the height of sand above and below seed ranged from 10-15 cm and 15-20 cm, respectively. They were watered at 4-5 day intervals after March. The germinated seeds were used as grafting rootstock after reaching stem height > 4 cm. In order to reduce the interference of the rootstock genotype, we adopted rootstocks of half-sib seed and scions of the same variety for grafting. After achieving grafting, the seedlings of different varieties were cultured in light media, which comprised of peat and perlite (2:1). Then, the seedlings were placed on the seedbed, and a sealed arch shed 50 cm high was built with a film to keep warm and moist during growth. After 60 days of grafting, the film was uncovered. After 210 days, we investigated the survival rate, took out the container seedlings of *C.oleifera*, and soaked them in water. After the media around the root system softens, we wash them with water and try to keep the root system intact. The residual water was absorbed by absorbent paper before measurement. The vernier caliper measured the ground diameter and height of seedlings. The characteristics of the root, including root length, root surface area, root volume, and root properties with different root diameters (0 < D1 ≤ 0.5 mm, 0.5mm< D2 ≤1.0 mm, 1.0mm< D3 ≤1.5 mm, 1.5mm< D4 *≤* 2.0* mm*), were determined by EPSON v700 dual light source special scanner. The scanning pictures were analyzed with the root image analysis software in WHIZO pro2020b. Collecting all the samples complies with institutional, national, or international guidelines and legislation. The local forestry management department authorized the collection of all samples for this research.

### Experimental treatments

Accurately weigh 1g sample into 2 mL centrifuge tube and add a grinding ball with a diameter of 6 mm; Add 400 μl extract (methanol: water = 4:1 (V:V)), containing 0.02 mg/mL internal standard (L-2-chlorophenylalanine); Frozen tissue grinder grinding for 6 min (-10°C, 50 Hz); Low-temperature ultrasonic extraction for 30 min (5°C, 40 KHz); The sample was placed at -20°C for 30 min; Centrifugation was performed for 15 min (13000 g, 4°C), and the supernatant was transferred to the injection vial with endocannula for machine analysis. In addition, remove 20 μL supernatant from each sample, mix and serve as quality control sample.

### Metabolite analysis

Metabolites in terminal buds of *C.oleifera* were extracted using 70% methanol and subjected to metabolomics analysis using an Ultra-Performance Liquid Chromatography Coupled With Electrospray Time-Of-Flight Mass Spectrometry (UPLC-Q-TOF/MS) (Waters, Milford, USA) at Majorbio BioPharm Technology Co., Ltd (Shanghai, China). Briefly, terminal buds were ground into a fine powder with liquid N_2_. Buds powder (50 mg) was extracted with methanol (1 mL, 70% aqueous) for 30 min in an ultrasonic bath. The extraction was maintained at -20°C for 20 min, followed by centrifugation at 13,000 × g at 4°C for 15 min. The supernatant was filtered using a microporous membrane (SCAA-104, 0.22-μm pore size, ANPEL, Shanghai, China) for LC-MS/MS analysis. Quality control (QC) samples were prepared by mixing sample extracts and injected every ten samples throughout the analytical run to provide data to assess repeatability. LC-MS was performed on a Waters UPLC I-class system equipped with a binary solvent delivery manager and a sample manager, coupled with a Waters VION IMS Q-TOF Mass Spectrometer equipped with an electrospray interface (Waters Corporation, Milford, MA, USA). Separation was performed using a Waters Acquity BEH C18 column (100 mm × 2.1 mm, 1.7 µm) (Waters Corporation, Milford, MA, USA). Mobile phase A was water containing 0.1% formic acid, and mobile phase B was acetonitrile containing 0.1% formic acid. The solvent gradient was as follows: T = 0, 5% B, ramped linearly to 20% B at 2 min, 60% B at 8 min; 100% B at 12 min, maintained at 100% B until 14 min, ramped linearly to 5% B at 14.5 min, maintained at 5% B until 15.5 min. The flow rate was 0.4 mL/min, sample volume 3 μl, and column temperature 45.0 °C. The eluate was passed into a Waters VION IMS Q-TOF Mass Spectrometer, equipped with an electrospray ionization (ESI) source operating in either positive or negative ion mode. The source and de-solvation temperatures were 120 °C and 500 °C, respectively, with a de-solvation gas flow of 900 L/h. Centroid data were collected from m/z 50–1000, with a scan time of 0.1 s and an interscan delay of 0.02 s over 13 min. Three independent extractions and analyses were performed.

Data filtering, peak detection, alignment, and calculations were performed with Waters Progenesis QI software (Waters Corporation, Milford, MA, USA), using the following parameters: Retention time range 0.5–14.0 min with tolerance of 0.01 min, mass range 50–1,000 Da with tolerance of 0.01 Da, noise elimination level was set at 10.00, minimum intensity was set to 15% of base peak intensity. Isotopic peaks were excluded for analysis. The excel file was obtained with three-dimensional data sets including m/z, peak RT and peak intensities, and RT-m/z pairs were used as the identifier for each ion. The resulting matrix was further reduced by removing any peaks with missing values (ion intensity = 0) in > 60% of samples. The internal standard was used for data QC (reproducibility). Principle component analysis (PCA) and orthogonal partial least squares-discriminant analysis (OPLS-DA) were carried out to visualize the metabolic differences between experimental groups after mean centering and unit variance scaling. Variable importance in projection (VIP) analysis ranked the overall contribution of each variable to the OPLS-DA model, and those variables with VIP > 1.0, *p*< 0.05, and fold change (FC) > 1 or< 1 were classified as differentially changed metabolites (DCMs). Qualitative analysis of each metabolite and its compound identification number acquisition was performed using the Human Metabolome Database (http://www.hmdb.ca/), followed by path annotations using the Kyoto Encyclopedia of Genes and Genomes (KEGG, https://www.genome.jp/kegg/pathway.html) database. Metabolite classification, significant metabolic pathway detection, and enrichment analysis were also carried out.

### Statistical analyses

Statistical analyses and graphical representations were performed using R version 4.2. Multivariate analyses, including PCA, and OPLS-DA, were carried out using the online platform of Majorbio Cloud Platform (www.majorbio.com) and pcaMethods (Stacklies et al., 2007) R packages. Multiple range tests were performed using least significant differences, and differences were considered significant at *p*< 0.05. Correlation analysis, heatmap, and network visualization were performed using the OmicStudio tools at (https://www.omicstudio.cn/tool).

## Results

### Growth of grafted seedlings

**Figure 2:**
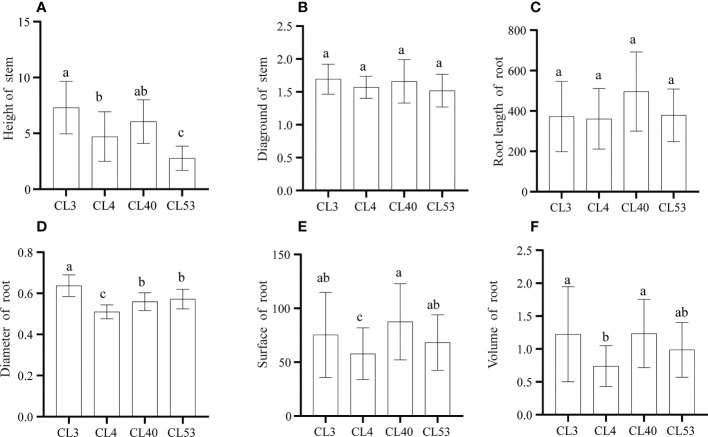
The growth of stem and root after grafting in C.oleifera. **(A)** Height (cm). **(B)** Diaground (mm). **(C)** Root Length (mm) **(D)** Root diamter (mm) **(E)** Root surface (mm2). **(F)** Root volume (mm3). Data represent the average of three replicates (n = 3) = standard error. Different letters indicate significant differences by Duncan's Multiple Range Test at p< 0.05. Height: seedling height. Diaground: ground diamter. Length: total root length. Surface: root surface. Avgdiam: average diameter.

There were significant differences in the growth of roots and stems among varieties after grafting. The survival rate of grafting was the highest in CL40. Although the grafting survival rate of CL3 was the lowest (60.87%) (**Supplementary Figure S1**), the height and ground diameter reached the maximum among varieties, and there were significant differences in seedling height between CL3 and CL4 and CL53 (**Figures 2A, B** **)**. As an essential index to measure seedling traits, the scion has a powerful regulatory effect on the root system (Long et al., 2013). The root length, root surface area, and root volume among varieties reached the highest in CL40 and the lowest in CL4, and there were significant differences between CL40 and CL4 in root surface area and root volume (**Figures 2C–F** **)**. This shows that there was a significantly different in root growth. Because root diameter is an indicator of root thickness, it is worth noting that after grafting, the proportion of D1 in CL4’s root length is 76.04%, CL40 65.52%, CL53 68.57%, and there was a significant difference between CL4 and other varieties (**Supplementary Figure S2A**). The proportion of D2 and D3 in root length reached a high value in CL3, and the lowest value was in CL4. The high growth of seedling propagation was the key to sapling, and it often chose a genotype with a high growth rate (**Supplementary Figures S2B, C**). However, the difference between varieties often led to the inconsistency between growth and grafting survival rates. For example, the 1-year-old seedling sapling rate of CL53 is shallow, which limits the promotion of this variety (Long et al., 2013). Therefore, it was necessary to evaluate the mechanism leading to this situation.

### Overview of metabolomics results

There were 5241 and 6235 detectable peaks in UPLC-MS positive and negative modes, respectively (**Supplementary Table S1**), after removing the peaks of internal standards and any known pseudo-positive peaks. The positive and negative data were combined into a data set with 9729 peaks (**Supplementary Table S2**), 833 metabolites of which were identified (**Supplementary Table S3**). In the principle components analysis (PCA) score plot, quality control (QC) samples are clustered together, suggesting this method has good stability and reproducibility. The contribution rate of the principal component of the two ions was 48.10% and 47.10%, respectively (**Supplementary Figures S3A, B** **)**, which could reflect the primary characteristic information of bud in *C.oleifera*. At the same time, the 6 biological repeat data of each of the 4 varieties were separated, indicating differences in species and content of metabolites among the cultivars. OPLS-DA was used to evaluate the difference in metabolites between quality. The results were shown the 12 groups of negative and positive ions, the R2X of each comparison group was more significant than 0.5, and the values of R2Y and Q2 were more significant than 0.9, indicating that the OPLS-DA model had an excellent fitting effect (**Supplementary Table S4**). It showed that the metabolites show a separation trend among varieties, suggesting that the metabolites in the buds had significant changes. 138 (17.31%) prenol lipids had achieved blast by HDMB databases (**Supplementary Table S3**). Further analysis of homologies had the highest homology with sequences from fatty acyls (12.30%), followed by organooxygen compounds (12.10%), carbolxylic acids derivatives (9.91%) (**Figure S3C**).

### Correlation analysis of metabolites-metabolites in regulation of metabolic network

**Figure 3:**
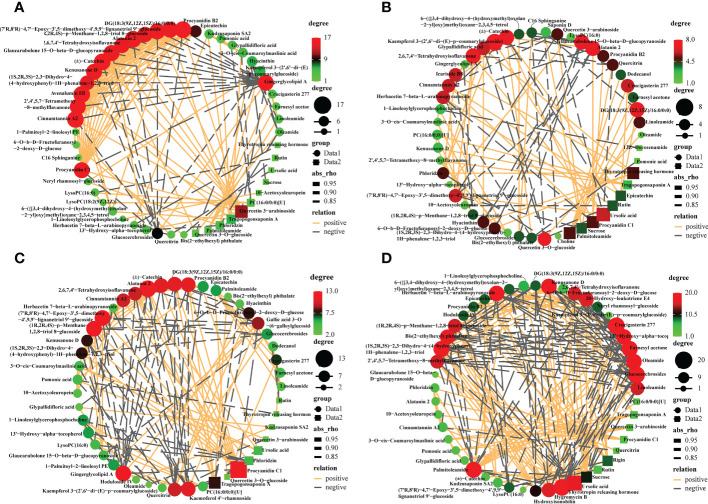
Metabolites -metabolites correlation within buds in different varieties. Positive and negative correlations are represented in orange and grey, respectively. Different color of nodes denotes distinct metabolites. **(A)** The interaction networks of 50 most abundant metabolites in the CL3. **(B)** The interaction networks of 50 most abundant metabolites in the CL4. **(C)** The interaction networks of 50 most abundant metabolites in the CL40. **(D)** The interaction networks of 50 most abundant metabolites in the CL 53.

To decipher the relationships between metabolites, we performed a correlation-based network analysis using significant pairwise correlations (r ≥ 0.5, *p<*0.05) (**Supplementary Table S5**). As expected, metabolites belonging to the same biochemical pathway tended to show a high degree of concatenation, with particular attention to the top50 most abundant metabolites for varieties. In total, significant correlations (r > 0.8) were detected 105, 136, 184, and 281 metabolites in CL3, CL4, CL40, and CL53, respectively, indicating that metabolite-metabolite correlations were different among varieties (**Figure 3**, **Supplementary Table S6**). The core metabolites differed among varieties. Hub metabolites in CL3 included Procyanidin B2 of flavonoids, Alatanin 2, (±)-Catechin, Avenalumin III, Cinnamtannin A2. The hub metabolites in CL4 were Kaempferol 3-(2’,6’-di-(E)-p-coumarylglucoside), followed by DG (18:3(9Z,12Z,15Z)/16:0/0:0), Alatanin 2, Glaucarubolone 15-O-beta-D-glucopyranoside, (±)-Catechin, 2,6,7,4’- Tetrahydroxyisoflavanone, Quercetin 3-O-glucoside. The hub metabolites in CL40 are Alatanin 2, Procyanidin C1, followed by (1R,2R,4S)-p-Menthane-1,2,8-triol 8-glucoside, gingerglycolipid A, procyanidin B2, DG(18:3(9Z,12Z,15Z)/16:0/0:0), (±)-catechin and kaempferol 4’-rhamnoside. The hub metabolites in CL53 include glucocerebrosides, crucigasterin 277 and thyrotropin releasing hormone, followed by farnesyl acetone, (1S,2R,3S)-2,3- Dihydro-4-(4-hydroxyphenyl) -1H-phenalene- 1,2,3-triol and palmitoleamide. Meanwhile, proanthocyanidin C1 was significantly positively correlated with cinnamon tannin A2 and alatanin 2 in four varieties. And cinnamontannin and alatanin 2 were significantly positive correlated between CL3, CL40, and CL53. It indicated that many flavonoids were not only the core metabolites of various varieties but also closely related to each other. It had been reported that procyanidins are formed by the polymerization of (±)-catechins or epicatechin phenolics (Wong-Paz et al., 2021). In this study, we found that there was also a significant positive correlation among (±)-catechins, procyanidin C1, procyanidin B2 in CL3 and CL40. And epicatechin in CL40 and CL53 had a significantly positively correlated with procyanidin B2. (±)-Catechin of CL4 had a significantly positively correlated with procyanidin C1, and procyanidin B2. The significant positive correlation between procyanidin C1 and procyanidin B2 and the significant positive correlation between procyanidin C1 and procyanidin B2 in CL4, indicated that there were differences among procyanidin metabolite species and (±)-catechins might affect the content of procyanidin C1. Epicatechin could regulate the important metabolites of procyanidin B2.

Polar auxin transport (PAT) was essential to control root growth (Zhang and Wang, 2021). Flavonoid metabolites, such as quercetin, rutin, apigenin, and kaempferol, act as a natural regulator of growth hormone transport that inhibit root development (Peer and Murphy, 2007; Peer et al., 2011; Yin et al., 2014), control the activity of growth hormone transport such as indole-3-acetic acid (IAA) (Brunetti et al., 2018; Zhang et al., 2022). Furthermore, substances that affect the activity of growth hormone oxidase balance the role of growth hormone metabolism (Brunetti et al., 2013). Intriguingly, (±)-catechins of CL4 were significantly negatively correlated with rutin, while CL53 was significantly positively correlated. (1R,2R,4S)-p-menthane-1,2,8-triol 8-glucoside, as an organic oxygen compound, was significantly positively correlated with cinnamon tannin A2 in CL3 and CL40 but significantly negatively correlated with CL53. Kaempferol had a significant positive correlation with root bark glycosides in CL3 and CL4, but CL40 was significantly negatively correlated; quercetin 3-arabinoside was significantly correlated with 2,6,7,4’-tetrahydroxyisoflavone, cinnamotannin A2 and (7’R, 8’R)-4,7’- epoxy-3’,5- dimethoxy-4’,9,9’-lignan triol 9, respectively ‘-glucoside was significantly positively correlated at CL3, while it was significantly negatively correlated at CL4. These results suggest that inter-metabolite correlations are influenced by the varieties, making the metabolic network more diverse, which may have different results on growth.

### Identification and functional classification of different metabolites

**Figure 4:**
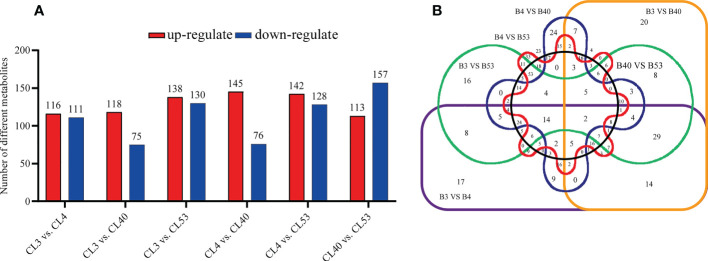
Comparative analysis of tissue-enrichaed and differentially metabolites. **(A)** Histogram showing the distribution of different metabolites between varieties. **(B)** Venn diagram showing the metabolites in buds compared to varieties.

Differential metabolites among varieties were filtered according to VIP > 1 and *p* value< 0.05. The number of different metabolites in CL4 *vs*. CL53 was minimum. There were much more metabolites in CL40 *vs*. CL53 and CL3 *vs*. CL53. Thus, CL53 might have more changes than CL3 and CL40, while the difference between CL4 and CL53 was small (**Figure 4A**; **Supplementary Table S7**). The up-down differential metabolites were screened according to the up-regulated fold change ≥ 1 and down-regulated fold change ≤ 1 (**Supplementary Table S8**). 145 metabolites in CL4 *vs*. CL40 were the highest to be up-regulated, and the lowest is 113 in CL40 *vs*. CL53. 157 in CL40 *vs*. CL53 metabolites was highest in down-regulated, and lowest is 75 in CL3 *vs*. CL40. The up-regulated metabolites in top20 include gibberellin A63, PS (18:3 (6Z, 9z, 12z)/20:1 (11z in CL3 and CL53, cafamarine, quercetin 3-o-glucosyl-rutinoside, (2S, 2’s) -pyrosaccharopine, (2R, 6x) -7-methyl-3-methyl-1,2,6,7-octanetrol 2-glucoside) in CL40 and CL53, zeranol in CL4 and CL53, and limonexic acid in CL40. The up-regulated metabolites in top20 include gibberellin A63, PS (18:3 (6Z, 9z, 12z)/20:1 (11z in CL3 and cl53, cafamarine, quercetin 3-o-glucosyl-rutinoside, (2S, 2’s) -pyrosaccharopine, (2R, 6x) -7-methyl-3-methyl-1,2,6,7-octanetrol 2-glucoside) in CL40 and CL53, zeranol in CL4 and CL53, limonexic acid and patuletin 3-gentiobioside in CL40. Down-regulated metabolites include monotropein, 2-o-protocatechuoylalphitolic acid, guaiacol, capsianoside I, goshonoside F1,10,20-dihydroxyeicosanoic acid in CL3, 1 α, two α, four β H,6 α, 8R) -p-menthane- 2,6,8,9-tetrol and kuwanon Q in CL4, and 2 (R) -hydroxydocosanoic acid in CL53 (**Supplementary Figure S4**).

In this study, the differential metabolites of each comparison group were analyzed by the Venn diagram (**Figure 4B**). Two kinds of differential metabolites were commonly expressed among varieties, including 2-phenethyl rutinoside and Hovenidulcioside A1 of carbohydrate and conjugate compounds. There were 29, 16, 16, and 53 specific metabolites in CL3, CL4, CL40, and CL53, respectively (**Supplementary Table S9**). The specific metabolites appeared in different varieties, including carboxylic acid and its derivatives and acrylol lipids in CL3, flavonoids, organic oxygen compounds, and propenol lipids in CL4, flavonoids, organic oxygen compounds, and acrylol lipids in CL40, and aliphatic acyl, flavonoid, propylene alcohol lipids, steroids, and their derivatives in CL53. Some of the separated differential metabolites had numbers of different metabolites, which allowed them to be annotated in the KEGG database. These pathways were mainly involved in the metabolism of cofactors and vitamins in CL3, global and overview maps and biosynthesis of other secondary metals in CL4, carbon metabolism, global and overview maps, lipid metabolism, biosynthesis of other secondary metabolites in CL53 (**Supplementary Table S9**).

**Figure 5:**
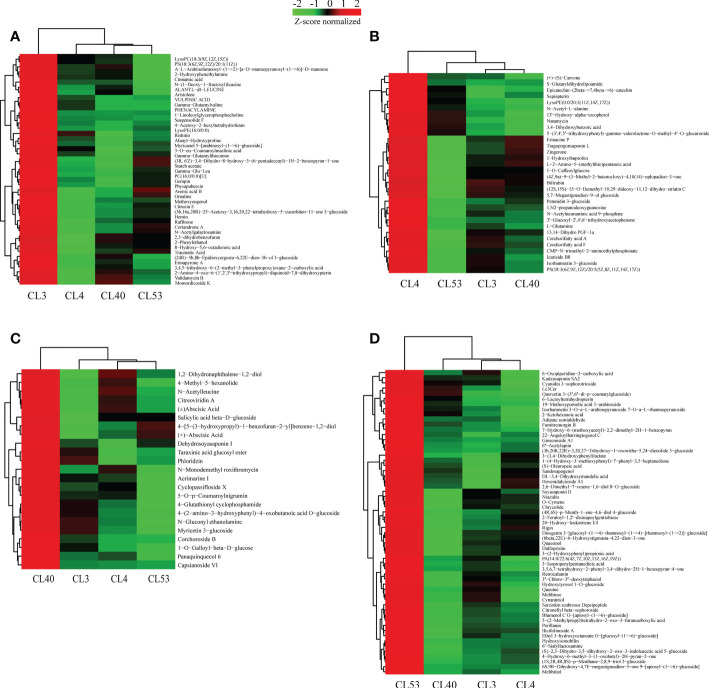
Relatively highly expreessed metabolites identified at a specific varieties. **(A)** Heatmap displaying the special expression meabolites in samples of CL3. **(B)** Heatmap displaying the special expression metabolites in samples of CL4. **(C)** Heatmap displaying the special expression meabolites in samples of CL40. **(D)** Heatmap displaying the special expression meabolites in samples of CL53.

In total, 554 metabolites were obtained from 6 groups of differential metabolites (**Figure 5A**, **Supplementary Table S8**). Judging from the proportion of differential metabolites in each class out of the total differential metabolites (**Supplementary Table S8**), the most active metabolites include prenol lipids, fatty acyls, carboxylic acids and derivatives, organooxygen compounds, flavonoids, steroids and steroid derivatives, benzene and substituted derivatives, glycerophospholipids, carbohydrates, and carbohydrate conjugates, and cinnamic acids and derivatives. These metabolites were grouped into superclasses of lipids and lipid-like molecules (208 metabolites), phenylpropanoids and polyketides (71 metabolites), organic acids and derivatives (22 metabolites), others (60 metabolites), organic oxygen compounds (53 metabolites), organoheterocyclic compounds (43 metabolites), benzenoids (26 metabolites), and others. At the subclass level, metabolites were further grouped into classes of prenol lipids (92 metabolites), fatty acids (41 metabolites), unknown metabolites (60 metabolites), carboxylic acids and derivatives (57 metabolites), organooxygen compounds (53 metabolites), flavonoids (38 metabolites), steroids and steroid derivatives (30 metabolites), Benzene and substituted derivatives (21 metabolites), Glycerophospholipids (16 metabolites), and others.

### Identification of specific metabolites among varieties

Metabolites play critical roles in various varieties. To provide insights into the regulatory network underlying buds of different varieties, we examined the expression of metabolites in different, especially their dynamic differentially expression. 554 metabolites belonging to 67 different classes were differentially expressed in the varieties examined (**Supplementary Table S10**). Analysis of the differentially expressed metabolites that were highly expressed between varieties (**Figure 5**, **Supplementary Table S11**) showed that 45, 32, 23, and 59 specific metabolites were highly expressed among varieties for CL3, CL4, CL40, and CL53, respectively, but there were significant differences in metabolite species between varieties. Most metabolites in the class level showed relatively broad expression patterns in all varieties, while some exhibited distinctive variety-specific patterns. Interestingly, metabolites of flavonoid were not found among the specific metabolites within CL3 (**Supplementary Table S12**). However, there were more number of amino acids, peptides, and analogs in CL3. Meanwhile, the number of flavonoid and prenol lipids in CL53 was more than in other varieties. The different KEGG pathways were obtained in specific metabolites (**Supplementary Table S11**). Three metabolic pathways were obtained in CL3: amino acid metabolism, transmembrane transport, and lipid metabolism. 9 metabolic pathways were in CL4, including global and overview maps, amino acid metabolism, other secondary metabolite biosynthesis pathways, and terpenoid and polyketone metabolism. CL40 had a global and overview diagram, and the metabolite was (+) -abscisic acid. CL53 obtained 7 metabolic pathways, including 2 global and overview maps, 2 amino acid metabolism, 1 transmembrane transport, 1 carbohydrate metabolism, and 1 lipid metabolism. CL53 had many metabolic pathways, reflecting the variety’s rich metabolic activities.

### Identification of metabolites associated with plant growth in grafting seedling

**Figure 6:**
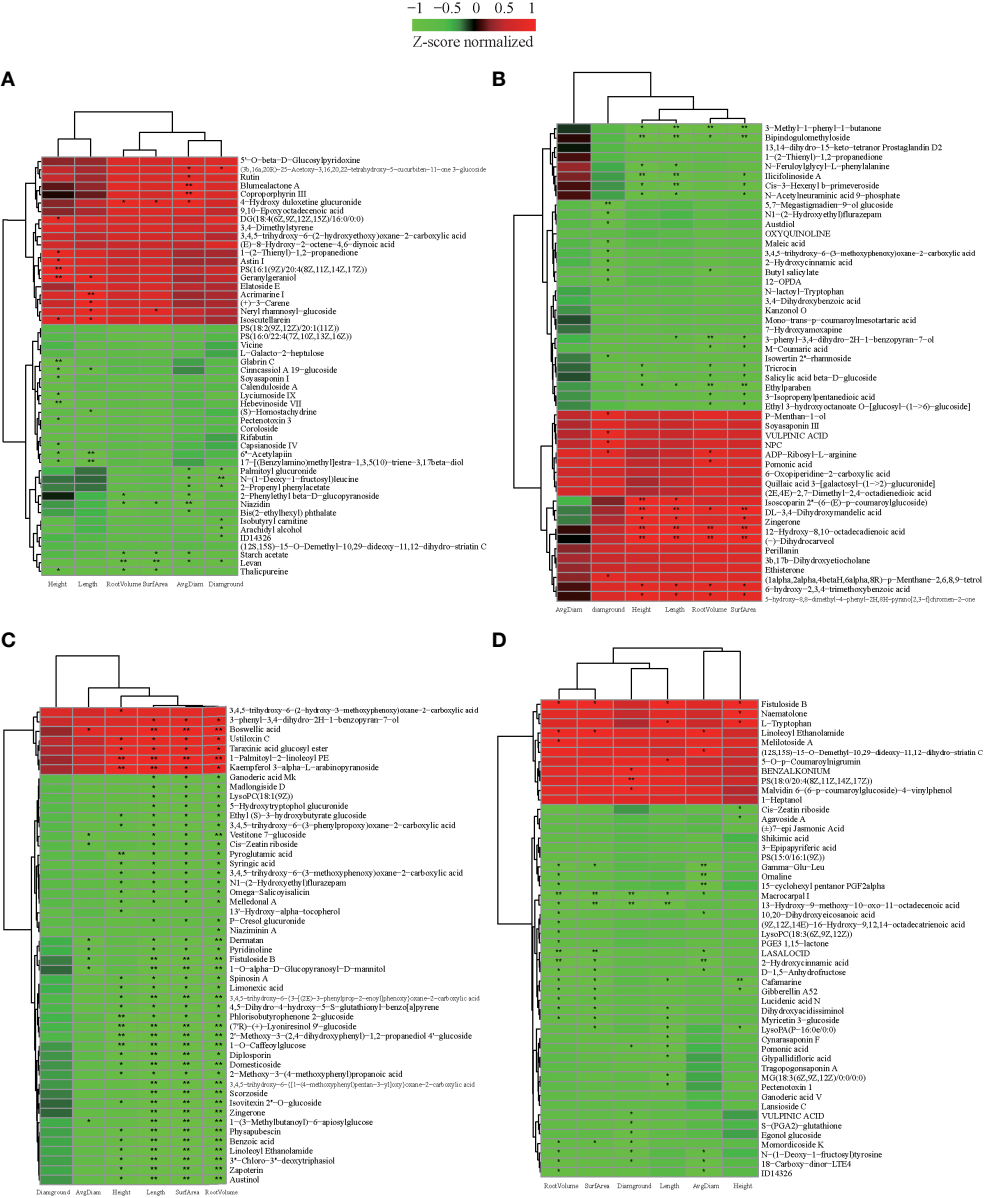
The correlation between metabolites and characteristics of the growth of the seedling. **(A)** The heatmap of top 50 expression abundant metabolites in CL3. **(B)** The heatmap of top 50 expression abundant metabolites in CL4. **(C)** The heatmap of top 50 expression abundant metabolites in CL40. **(D)** The heatmap of top 50 expression abundant metabolites in CL53. Height: seedling height. Diaground: ground diamter. Length: total root length. Surface: root surface. Avgdiam: average diameter. *. Significantly correlated at the P < 0.05 level; **. Highly significantly correlated at the P < 0 .01 level.

We also investigated whether the metabolic composition of cultivars would impact seedling growth after grafting. In order to determine the types of critical metabolites that affect the growth of grafted seedlings, the known metabolites obtained from each variety were analyzed for correlation with the growth characteristics of grafted seedlings (|r| > 0.5, *p*< 0.05), and the data were screened according to |r| > 0.8. The 50 metabolites with the highest correlation were used to construct the correlation heat map (**Figure 6**, **Supplementary Table S13**). There were 36, 35, 50, and 42 metabolites in CL3, CL4, CL40, and CL53, respectively. It had a common significant and highly significant correlation with the growth of grafted seedlings. At the same time, 11, 16, 30, and 9 metabolites of CL3, CL4, CL40, and CL53 were significantly or very significantly correlated with root and stem characteristics, while they were significantly correlated with stems 9, 11, 2, and 9 metabolites, respectively. There were significantly correlated with rooting in 3, 4, 10, and 25 metabolites, respectively. These indicated that the regulation of the metabolites in the bud on the grafting growth was significantly different among varieties. Because phenols (anthocyanins, flavanones, p-coumaric acid, and hydroxybenzoic acid) and (±)catechins have a significant effect on grafting (Hudina et al., 2014; Dinc et al., 2018; Elsheery et al., 2020; Gamba et al., 2021), we hypothesized that the metabolic composition of the buds could be one of the key factors determining seedling growth.

We further analyzed the role of these metabolites in plant growth. (±)-Cafamarine and gibberellin A_52_ negatively regulate seedling height and root growth. In particular, 6’’-acetylapiin, 17-[(benzylamino) methyl] estra-1,3,5 (10)-triene-3, 17beta diol, cinncassiol a 19-glucoside in CL3 are significantly negatively correlated with stem and root length. Moreover, geranylgeraniol was very significantly and significantly positively correlated with seedling height and root length, respectively. Acrimarine I, (+)-3-CARENE, (s)-homostachydrine and root length have extremely significant, significant positive, and negative correlations. Niazidin, levan, starch acetate, and 4-hydro duloxetine gluconide had significant negative effected on root traits. Dl-3,4-dihydroxymandelic acid, zingerone, 12-hydroxy-8,10-octadecadienoic acid, (-) -dihydrocarveol of CL4 had a very significant positive correlation with seedling height, root length, and root surface area. In contrast, ethylparaben, 3-methyl-1-phenyl-1-butanone, and bipindochloromethylide had a significant or extremely significant negative correlation and are very significantly correlated with root length. As a flavonoid, it regulated the transportation of this auxin, Geranylgeraniol was significantly positively correlated with the growth of stem segment and root length. Boswellic acid of CL40 was significantly or extremely significantly positively correlated with root growth traits, while vestitone 7-glucoside, cis-zeatin riboside, dematan, pyredinoline, fistuloside B, 1-o-alpha-D-glucopyranosyl-D-mannitol, 1-(3-methylbutanoyl)- 6-apiosyl glucose negatively regulates root growth. The metabolites, including phyllisobutyrophenone 2-glucoside, (7’r)-(+)-lyoneresinol 9’-glucoside, 2’-methoxy-3- (2,4- dihydroxyphenyl)-1,2-propanediol 4’-glucoside, 1-o-caffeoylglucose, pyroglutamic acid, negatively regulated height of seedling. 1-palmitoyl-2-Linoleoyl PE and kaempferol 3-alpha-L- arabinopyranoside regulated seedling height growth. Many glycosides were closely related to seedling height and root growth. The metabolites in CL53, including macrocarpal I, 13 -hydro-9-methoxy-10-oxo-11-octadecenoic acid, had a significant or extremely significant negative correlation with root and ground diameter. Furthermore, fistuloside B, cafamarine were regulated root and seedling height. This showed obvious differences in the metabolites regulating rhizome growth among varieties. The metabolites might be more focused on regulating the growth of stems in CL3 and CL4, rhizome in CL40, and roots in CL53.

Interestingly, zingerone had a significant positive correlation with seedling height, root length, and root surface area in CL4, while it had a very significant negative correlation with the root in CL40. Linoleoyl ethanolamide had a significant positive correlation with root surface area, root volume, and average root diameter in CL53. In contrast, it had a very significant negative correlated with root length, root volume, and average root diameter and a significant negative correlation with seedling height in CL40. This indicated that the same metabolites had a differentiation effected among varieties. Cis-zeatin riboside negatively regulated root growth in CL40 and negatively regulated stem growth in CL53. Because cis-zeatin riboside could regulate cell growth, root hair growth, and length of root formation, it was not conducive to the content of phosphorus in buds (Peer et al., 2011; Silva-Navas et al., 2019). Under the condition of phosphorus deficiency, cis-zeatin was necessary for root hair elongation and phosphorus distribution (Silva-Navas et al., 2019), and CL53 grows slowly after grafting, indicating that this metabolite may play an essential role in stem growth.

## Discussion

### Metabolite differences among varieties affect growth of root and stem in seedlings

The interaction between scion and rootstock will increase the xylem microtubule system to promote growth and affect rootstock’s water movement and photosynthesis process, while scion mainly affects the fruit’s physical and chemical quality characteristics of fruit (Zaaroor-Presman et al., 2020; Reyes-Herrera et al., 2020; Naik et al., 2021; Cañas-Gutiérrez et al., 2022). The scion of variety affects the growth and development of rootstock through various stimulation signals. These signals regulated plant root elongation and development (Gautier et al., 2019). In *Citrus reticulata*, the overall distribution of 6 out of 53 in scion and 14 out of 55 in rootstock basic metabolites was controlled by rootstock, whereas 42 and 33 were affected by the rootstock-scion interaction, correspondingly (Tietel et al., 2020). Metabolites at the grafting union of grape rootstock affected the growth and variation after grafting (Prodhomme et al., 2019) and bacterial diversity depending on the rootstock combination, which was affected by the identity of scion varieties and rootstocks and was mainly clustered according to variety differences (Vink et al., 2021). It could be assumed that the metabolites contained in scions might play a key partner role in the growth after grafting under the mechanism of rootstock scion interaction.

Therefore, our study used metabolomics based on ultra-high performance liquid chromatography-mass spectrometry to reveal the differences in buds between different genotypes. The metabolic expression profiles of leaf buds of *C.oleifera* after 30 days of sprout were analyzed, and the changes of metabolites among the four varieties were evaluated. It was found that there were significant differences in metabolites in buds among cultivars. Key metabolites and pathways regulating plant graft healing and root growth were obtained through analysis of metabolite and KEGG. These metabolites were involved in graft union, root growth, and signal transduction. Similar to the grafting process involving differences in bud metabolites of other species, many different metabolites have been identified to participate in various metabolic pathways and growth processes. Most differential metabolites were related to resistance regulation, followed by root growth. This reflected vigorous physiology and genetic regulatory activities in the bud. Each variety had its unique metabolites, and these factors may make the growth of new shoots and roots significantly different after grafting. These results indicated that a certain proportion of metabolites in buds may play an essential role in controlling the growth of *C. oleifera* after grafting.

### Metablite-metablite correlation provide new insights on the regulation of metabolic networks

In order to further unravel differences in varieties, a metabolite–metabolite correlation analysis was performed. In this study, there were more metabolites in CL53 with significant correlation but noticeable differences among varieties in core metabolites. The core metabolites of CL3, CL4, and CL40 all contain flavonoid metabolites that play an essential role in plant growth, development, stress resistance, and cell differentiation, such as proanthocyanidins, (±)-catechins, quercetin, and kaempferol (**Supplementary Table S6**), and are highly correlated as a whole. In particular, (±)-catechin, kaempferol, and coumarin are the main substances that cause incompatibility between scion and rootstock (Assunção et al., 2019). Catechin is often considered a marker metabolite of graft compatibility, and its content is affected by light, nitrogen nutrition, and other factors. The increase of sufficient ${\text{NO}}_{3}^{-}$ could affect the accumulation of (±)-catechin, while nitrogen deficiency will affect plants’ biosynthesis of anthocyanin-derived flavonoids (Li et al., 2019). This indicates that the vigorous growth of scions may benefit grafting and growth (Long et al., 2016). In this study, it was interesting that rutin and catechin are significantly negatively correlated in CL4 and positively correlated in CL53. It was reported that CL4 grew vigorously after grafting, while CL53 grew slowly. Rutin was a metabolite that inhibited IAA transport (Hong et al., 2005). The close relationship between (±)-catechin and rutin indicates that rutin may affect the grafting and growth of CL53. In addition, glucocerebroside and cruciflorin 277, the hub metabolites of CL53, can enhance plants’ stress resistance and antibacterial effect. In particular, glucocerebroside was the only glycosphingolipid found in plants, fungi, and animals. Moreover, it was one of the most abundant glycosphingolipids in plants, which plays an essential role in improving CL53 resistance. Our data showed a differential relationship between the metabolites after the development and maturation of buds. The interaction of various specific metabolites enables the scion to resist the effects of various adversity. The buds might be not only the guide of growth but also the guide of grafting and growth of scion.

### Metabolic composition is important for grafting seedling performance

Because plants usually assimilate nitrogen (N) into Glu, Gln, ASP, and Asn (Fisher et al., 1998), tea mainly transports Gln, theanine, and Glu from roots to branches (Oh et al., 2008). Glutamate is the center of amino acid metabolism, suggesting the role of glutamate as a signal molecule for sensing nitrogen status and controlling this process (Ohashi et al., 2015). In addition, glutamate is a precursor of serine biosynthesis in the non-photorespiratory pathway that can control the phosphorylation pathway of cell proliferation (Ros et al., 2014; Igamberdiey and Kleczkowski, 2018). In this study, the specific metabolites of amino acids, such as glutamic acid of CL3, were significantly more than those of other varieties. Moreover, the growth of CL3 after grafting was better than that of other varieties. It may be closely related to this variety’s relatively strong N transport capacity. ABA acts as a critical hormone to regulate stress tolerance, root growth, and dormancy in buds (Xu et al., 2020; Sun et al., 2018; Xie et al., 2019; Miao et al., 2021; Pan et al., 2021). In this study, CL40 has much more root length and grafting survival rate, and the specific metabolite of ABA in CL40 indicates that it may significantly promote root growth.

These results indicated that the bud’s specific metabolites might significantly affect the growth after grafting. Therefore, we further analyzed the correlation between metabolites and characteristics. Gibberellin was often found in *Arabidopsis* and rice to inhibit lateral bud germination and root growth (Qin et al., 2022). In this study, the apical bud with apical dominance was selected as the experimental material (Zhuang, 2008), and various varieties had a negative regulatory effect on the seedling height and root length after grafting, indicating that GA_52_ may be the primary metabolite that inhibits lateral bud germination and root growth. Because cs-Zeatin nucleoside could regulate cell growth, root hair growth, and long root formation (Silva-Navas et al., 2019). In this study, the root length of CL40 was the highest, and the seedling height of CL53 was lower among the varieties, indicating that the metabolite may play a role in promoting root growth and inhibiting shoot growth in the two varieties. These data indicated that the metabolite components in the bud had a significant impact on the growth after grafting, indicating that various metabolites might systematically promote the growth of grafted *C.oleifera* seedlings and produce differential growth performance.

These results indicated that the scion was involved in the growth of the root, and the scion’s communication between the root and soil may be affected. At the same time, tree growth and fruit yield depend on the supply of sufficient water, mineral nutrients, and other substances, which require the absorption of roots and the defense against pests and diseases. Moreover, various stable ecological chains have been broken with increasingly severe climate problems and environmental damage. Forestry, plagued by drought, water shortage, barren, pests, and diseases, would face more severe biological and abiotic stress, especially in the woody oil industry. Therefore, roots with vigorous growth and strong disease resistance could promote tree growth, yield, and environmental adaptability. Consequently, it was necessary to analyze further the mechanism of scion regulating root and explore the interaction effect between root and soil under the mechanism of rootstock scion interaction, especially the changes in tree growth and fruit yield caused by the influence of scion on the composition of root exudates on rhizosphere microbial community and species. This will help us to take targeted cultivation measures and improve the management level.

## Conclusion

Early research found that the scion of *C.oleifera* had a significant regulatory effected on the properties of rootstock after grafting and significantly impacted the growth of grafted seedlings. The metabolome was considered a powerful tool for connecting phenotype-genotype interactions, which impacted plant growth and development. In this study, four varieties of *C.oleifera*, planted in many areas in south China, were selected to analyze the differences in metabolites of the bud of scions and their effects on seedling characteristics after grafting. It explored the metabolite types and metabolic networks that may regulate the growth after grafting in buds. The analysis showed that each variety had a unique metabolite type and correlation network relationship. Differentiated metabolites showed different developmental trends after grafting. Many metabolites regulated the growth of roots and stem in buds before grafting, which played a crucial role in the growth of seedlings after grafting. It not only regulated the growth of roots but also affected the development of this stem segment. Finally, these results were associated with the genetic background of each variety, indicating that metabolites could potentially be used as indicators for seedling growth characteristics. To sum up, this study will enrich the theoretical basis of seedling growth and lay a foundation for further research on the molecular regulation mechanism interaction between rootstock and scion, rootstock growth, and development of grafted seedlings after grafting.

## Data availability statement

The original contributions presented in the study are included in the article/**Supplementary Material**. Further inquiries can be directed to the corresponding author.

## Author contributions

WL designed the experiment, performed data processing and drafted the manuscript. KW helped in bioinformatics analysis and data interpretation. GH and LL prepared the materials and performed the experiments. CY and KW participated in the design to the study, helped in data processing, and revision of the manuscript. GH and XY assisted in results interpretation and manuscript preparation. WL and KW conceived the study and revised the manuscript. All authors contributed to the article and approved the submitted version.

## Funding

This research was funded by Zhejiang Science and Technology Major Program on Agricultural New Variety Breeding (2021C02038); National Key R&D Program of China (2019YFD1001602); Quzhou Science and Technology project (2020K31).

## Acknowledgments

Data summarized in this paper have been generated through work of several authors and we would like to thank them for their continuous efforts which contribute to the emergence of the *Camellia oleifea*.

## Conflict of interest

The authors declare that the research was conducted in the absence of any commercial or financial relationships that could be construed as a potential conflict of interest.

## Publisher’s note

All claims expressed in this article are solely those of the authors and do not necessarily represent those of their affiliated organizations, or those of the publisher, the editors and the reviewers. Any product that may be evaluated in this article, or claim that may be made by its manufacturer, is not guaranteed or endorsed by the publisher.

## Supplementary material

Supplementary Table 1 The number of raw metabolites.

Supplementary Table 2 The number of metabolites.

Supplementary Table 3 The 833 known metabolites among varieties.

Supplementary Table 4 Parameters of OPLS-DA analysis.

Supplementary Table 5 The correlation of 834 metabolites in different varieties.

Supplementary Table 6 The correlation of metabolites in top 50 abundant metabolites.

Supplementary Table 7 The different metabolites in each comparison.

Supplementary Table 8 Number of different metabolites (up or down regulated) among varieties.

Supplementary Table 9 The common metabolites in comparative varieties by Venn diagram.

Supplementary Table 10 The different metabolites among varieties.

Supplementary Table 11 The specific metabolites among varieties.

Supplementary Table 12 The catalog of different metabolites among varieties.

Supplementary Table 13 The metabolites of correlation between metabolites and characteristics of seedling.
