# Supplementary figures and images for: Untargeted metabolism approach reveals difference of varieties of bud and relation among characteristics of grafting seedlings in Camellia oleifera
Source: Front Plant Sci. 2022 Nov 21;13:1024353. doi: 10.3389/fpls.2022.1024353 (PMC9720148; doi:10.3389/fpls.2022.1024353)

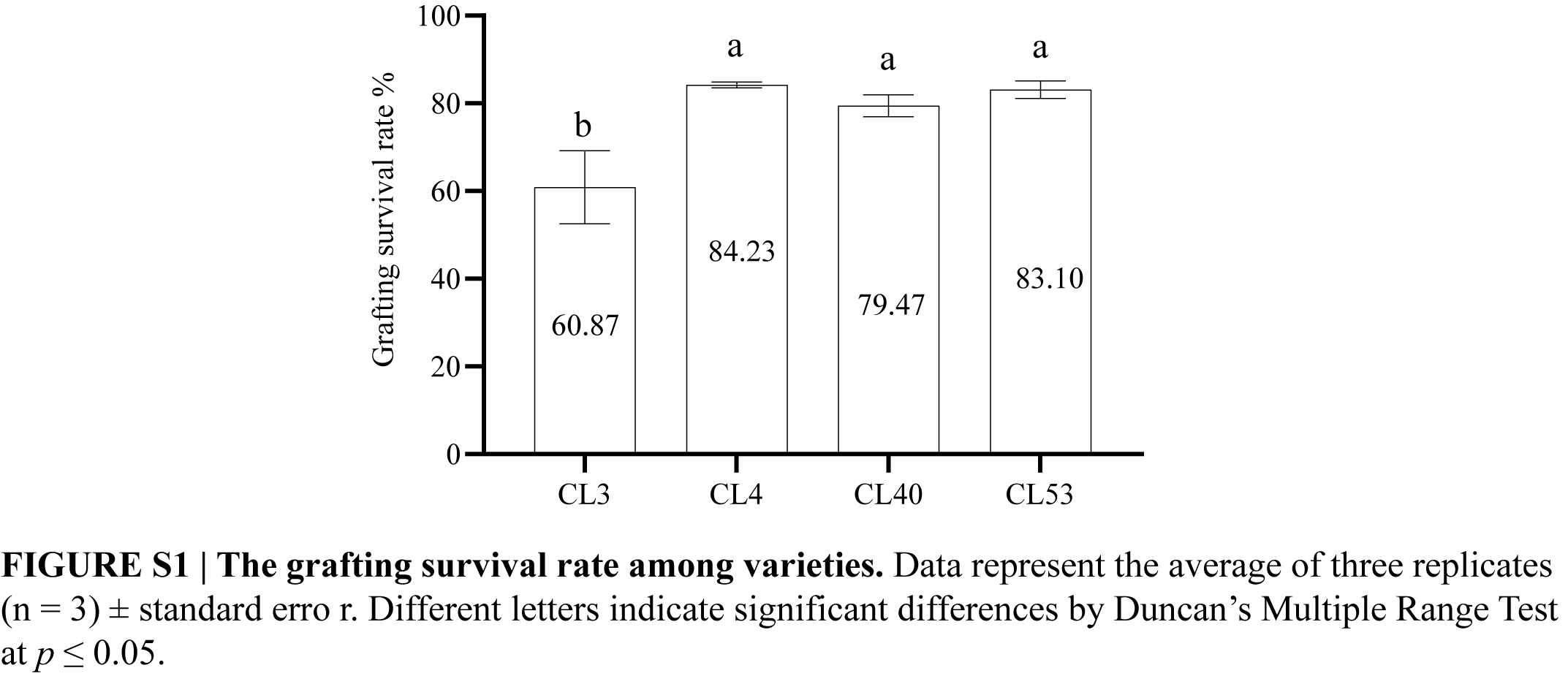

Supplement: Supplementary file 14 [file Image_1.tif]

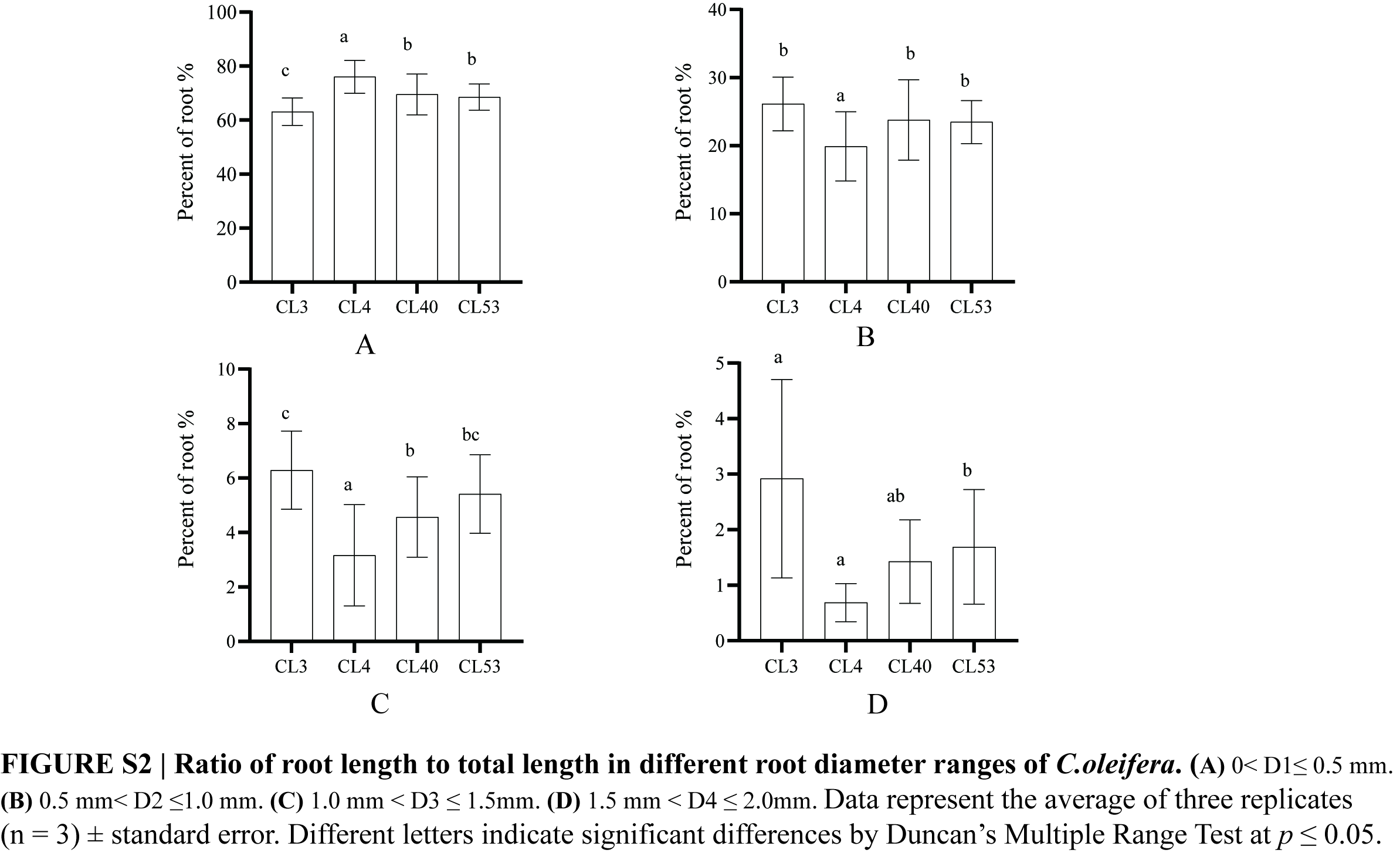

Supplement: Supplementary file 15 [file Image_2.tif]

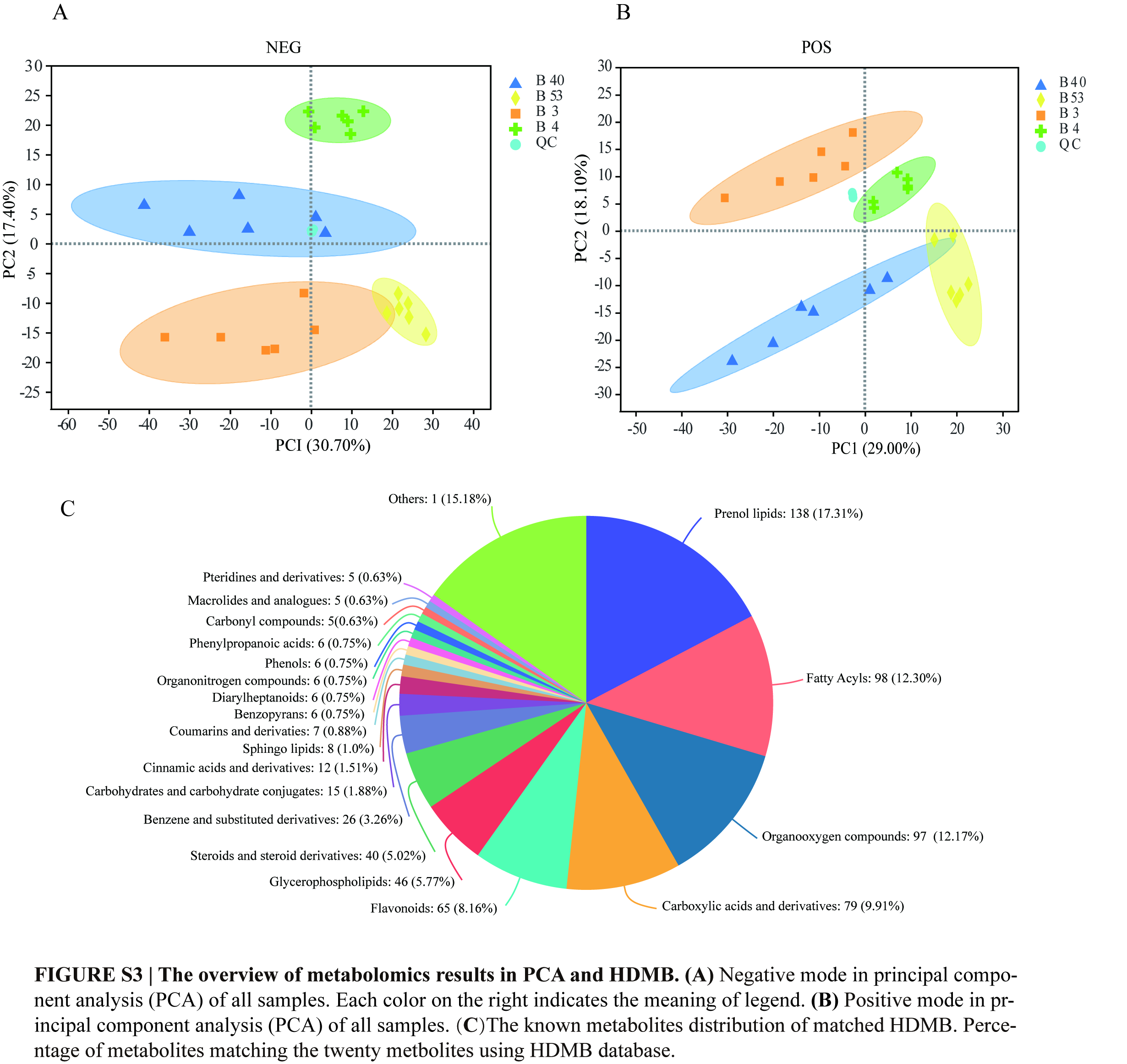

Supplement: Supplementary file 16 [file Image_3.tif]

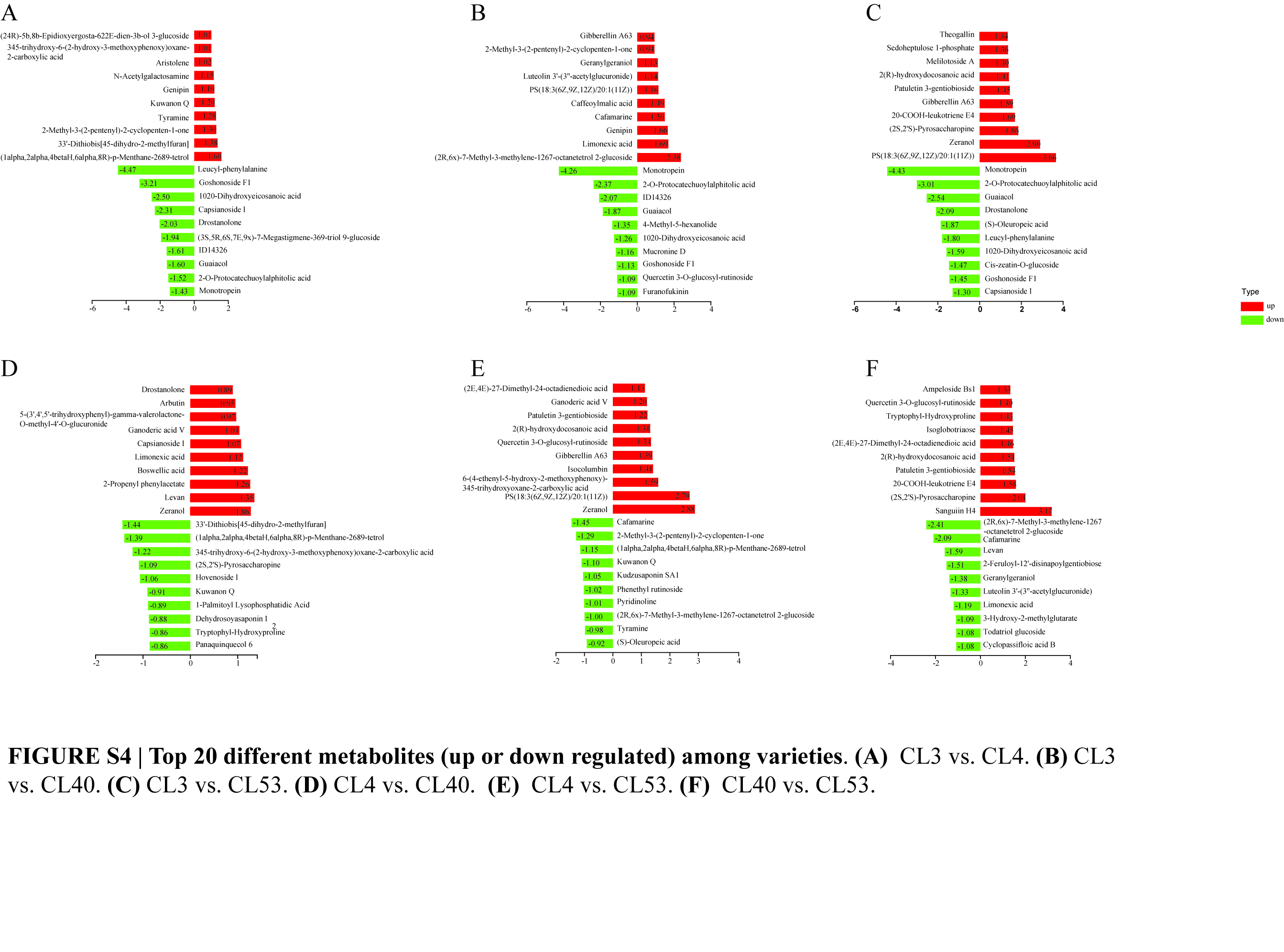

Supplement: Supplementary file 17 [file Image_4.tif]
